# Surmounting the summit: tackling cerebral malperfusion in AMDS-treated acute deBakey I dissections. A case report

**DOI:** 10.3389/fcvm.2024.1429287

**Published:** 2024-09-26

**Authors:** Nunzio Davide de Manna, Florian Helms, Ezin Deniz, Till Frederik Kaireit, Omar Abu-Fares, Bastian Schmack, Arjang Ruhparwar, Alexander Weymann, Aron Frederik Popov

**Affiliations:** ^1^ Department of Cardiothoracic, Transplant and Vascular Surgery, Hannover Medical School, Hannover, Germany; ^2^ Department of Diagnostic and Interventional Radiology, Hannover Medical School, Hannover, Germany; ^3^ Department of Diagnostic and Interventional Neuroradiology, Medical School Hannover, Hannover, Germany

**Keywords:** acute aortic dissection, ascyrus medical dissection stent, cerebral malperfusion, stroke endovascular treatment, dissected aorta repair through stent implantation

## Abstract

**Background:**

Acute Type A aortic dissection (ATAAD) with supra-aortic branch (SAB) malperfusion remains a formidable clinical challenge, often resulting in high mortality and complex treatment dilemmas. The introduction of the AMDS represents a significant innovation, designed to stabilize the aortic arch, and manage malperfusion effectively.

**Methods:**

This case study evaluates the utility of AMDS in the treatment of a 63-year-old male with hypertension, who presented with severe, acute chest pain. Diagnosed with a DeBakey type I ATAAD involving SAB, the patient underwent cardiopulmonary bypass, aortic root replacement, aortic arch repair with AMDS implantation, and subsequent endovascular stenting for severe left common carotid artery malperfusion that developed postoperatively. The AMDS was instrumental in facilitating crucial aortic arch reconstruction and addressing the initial severe malperfusion. Despite postoperative cerebral malperfusion, targeted endovascular stenting resulted in a rapid and substantial neurological recovery. The patient was discharged to a rehabilitation facility on postoperative day 20, free of neurological deficits.

**Conclusions:**

The use of AMDS in managing ATAAD with SAB involvement is transformative, enabling less invasive surgical techniques and offering immediate, effective correction of malperfusion. This case underscores the essential role of integrating advanced endovascular strategies to enhance outcomes in high-risk aortic surgeries, marking a pivotal advancement in the therapeutic approach to complex aortic dissections**.**

## Introduction

Acute type A aortic dissection (ATAAD) with supra-aortic branch (SAB) malperfusion presents with daunting mortality risks, often leading to compromised organ function and neurological impacts (1, 2). Addressing SAB malperfusion remains complex, even with advances in aortic surgery. Moreover, the strategy targeting the aortic arch is a matter of concern, especially in patients with no intima tear within the arch region (3). Surgical amelioration of SAB malperfusion at the point of recognition is the gold standard but often proves impractical, whereas techniques such as surgical or percutaneous fenestration have been efficacious for relieving malperfusion in the visceral, renal, and peripheral arteries.

The Ascyrus Medical Dissection Stent (AMDS) marks a paradigm shift in the treatment of malperfusion syndromes (4), offering a new avenue for positive aortic arch remodeling while aligning with primary surgical procedures (5).

The new hybrid graft is designed for DeBakey I dissections with a primary entry tear in the ascending aorta and no tears in the aortic arch or proximal descending aorta. The stent is available in two configurations: tubular, with a uniform diameter, and tapered, with a decreasing diameter from proximal to distal. Preoperative CT scans using multiplanar or center-line reconstructions determine sizing at two aortic landmarks: zone 1 of the aortic arch and the descending aorta at the tracheal bifurcation. The AMDS integrates a proximal Teflon fabric graft with a specialized Nitinol frame, ensuring low chronic outward force, high kink resistance, and adaptability to the aortic arch's curvature. It offers extensive thoracic aortic coverage to mitigate malperfusion and features a large open-cell structure for unobstructed perfusion of major aortic branches. The stent facilitates re-intervention of aortic branch vessels and distal re-entry tears when necessary. As an adjunct to standard surgical repair, the AMDS enhances malperfusion management and promotes favorable remodeling of the aortic arch and distal dissected aorta during initial surgery, without significantly extending or complicating the standard procedure in critically ill patients (6).

We present a novel case where AMDS-enabled endovascular stenting effectively remediated a postoperative cerebrovascular event caused by severe malperfusion in the left common carotid artery (LCCA) following an acute type A aortic dissection (ATAAD). This case signals a pivotal progression in acute aortic dissection management strategies even when the AMDS was implanted.

## Case description

**Figure 1:**
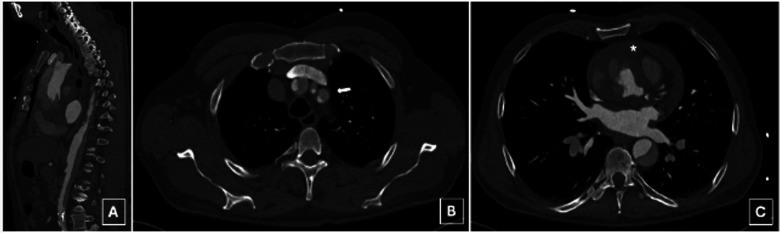
A preoperative computed tomography angiography disclosed an acute deBakey type I aortic dissection extending from the aortic root to the external iliac arteries **(A)**, including supra-aortic branch (SAB) involvement **(B**, arrow**)** and hemorrhagic pericardial effusion **(C**, asterix**)**.

**Figure 2:**
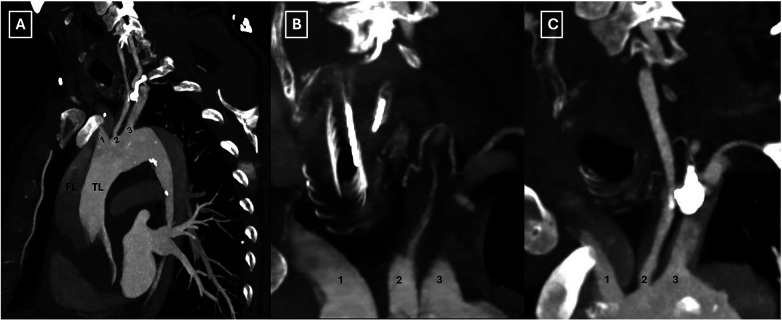
Preoperative computed tomography scan illustrating the involvement of all supra-aortic branches **(A–C)**. TL: true lumen; FL: false lumen; 1: innominate artery; 2: left common carotid artery; 3: left subclavian artery.

A 63-year-old male, with a history of hypertension, presented to the emergency department with acute, severe, and migratory chest pain of a few hours’ duration. On evaluation, the patient was alert with no neurological deficits, and the physical examination was otherwise noncontributory. A computed tomography (CT) angiography disclosed an acute DeBakey type I aortic dissection extending from the aortic root to the external iliac arteries, including supra-aortic branch (SAB) involvement and hemorrhagic pericardial effusion, while visceral arteries remained unremarkable, however with left kidney malperfusion syndrome (Figures 1A–C, 2A–C).

Initiation of cardiopulmonary bypass (CBP) was via direct arterial cannulation of the right axillary artery and venous return from the right atrium, conducted under moderate hypothermia (28°C). After aortic cross-clamping, aortic root inspection was conducted post-administration of Bretschneider's crystalloid cardioplegia (Custodiol®). With the esophageal temperature at 28°C, antegrade unilateral cerebral perfusion was instituted following clamping of the innominate artery. The ascending aorta was excised to the predestined anastomosis site at zone 0 of the aortic arch, which, upon exploration, displayed no entry tears. An AMDS 55/40 mm tapered (AMDS, ARTIVION, Kennesaw, USA) was placed via antegrade implantation during hypothermic circulatory arrest (HCA, 28 min) into the aortic arch and the descending thoracic aorta. The Device was positioned within the true lumen (TL) of the aortic arch and the descending thoracic aorta. Distal anastomosis between the aorta and the AMDS collar was performed using a new Dacron tube reinforced by an external Teflon felt. CPB was then re-initiated, the aortic arch was purged, the Dacron tube was clamped, and rewarming commenced until normothermia was achieved. At this stage, the anastomosis between the aortic arch, AMDS, and the ascending aortic graft was completed, as well as the proximal bypass graft anastomosis.

**Figure 3:**
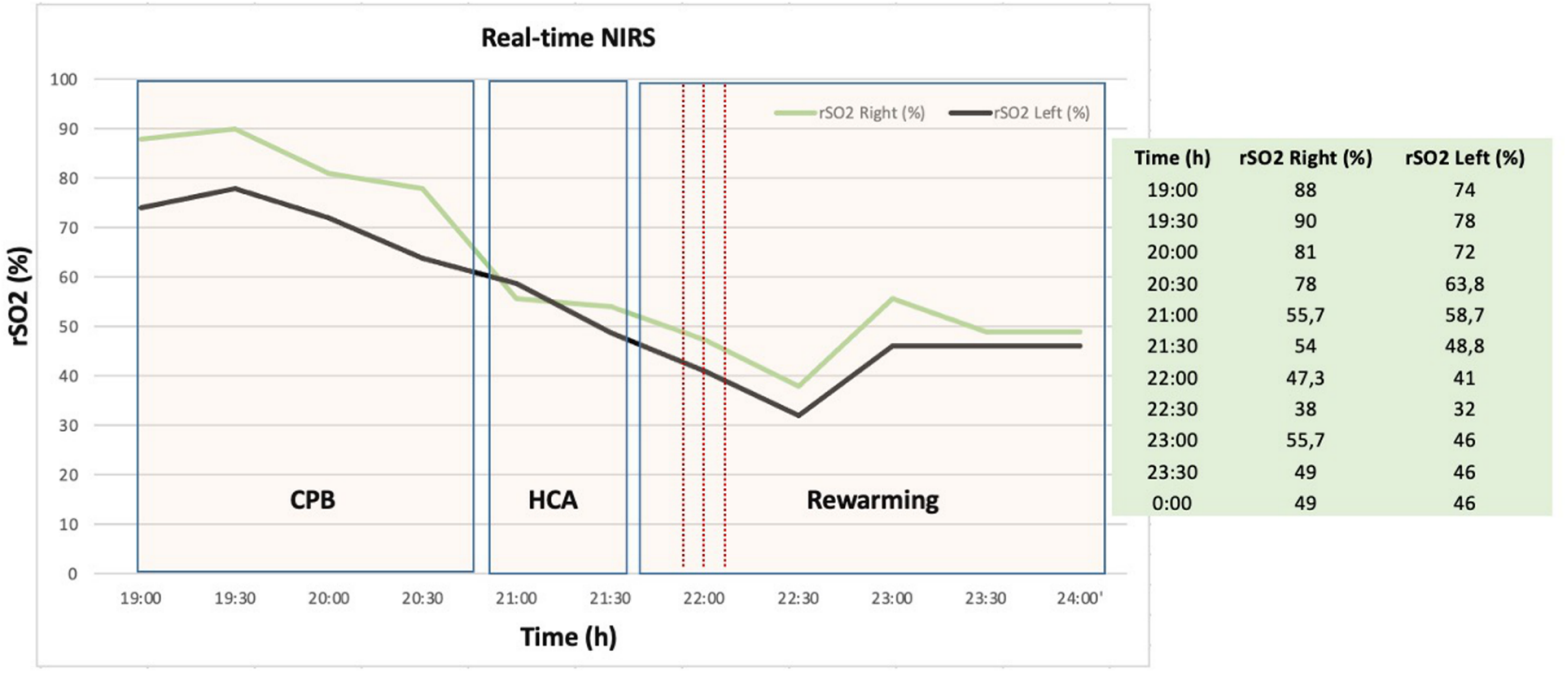
Representative example of near infrared spectroscopy (NIRS) measurement during aortic arch repair procedure with corresponding operation phases. Real-time NIRS graphic shows rapid decline of regional cerebral oxygen saturation (rSO2) before the onset and after cardiac arrest due to cerebral malperfusion. CPB, cardiopulmonary bypass; HCA, hypothermic circulatory arrest; rSO2, regional cerebral oxygen saturation; NIRS, near infrared spectroscopy; h, hours.

Intraoperatively, the primary entry tear was identified at the aortic root, involving the right coronary ostium. This discovery prompted us to undertake an aortic root replacement (Bentall procedure) employing an On-X Aortic Valve 25 mm (On-X® Life Technologies, Inc.) and 30 mm × 10 cm Gelweave Valsalva Graft (Terumo Cardiovascular Systems Inc, Ann Arbor, Mich) accompanied by a right coronary artery bypass. CPB weaning ensued after a duration of 172 min on inotropes and vasopressors, with cerebral perfusion lasting 28 min and aortic cross-clamp time at 128 min. During HCA, a rapid decline in bifrontal regional oxygen saturation (rSO2) was noted. This was followed by a slight increase during the rewarming phase (Figure 3). Unilateral antegrade cerebral perfusion was maintained via right subclavian artery perfusion at a rate of 8–10 ml/kg/min.

**Figure 4:**
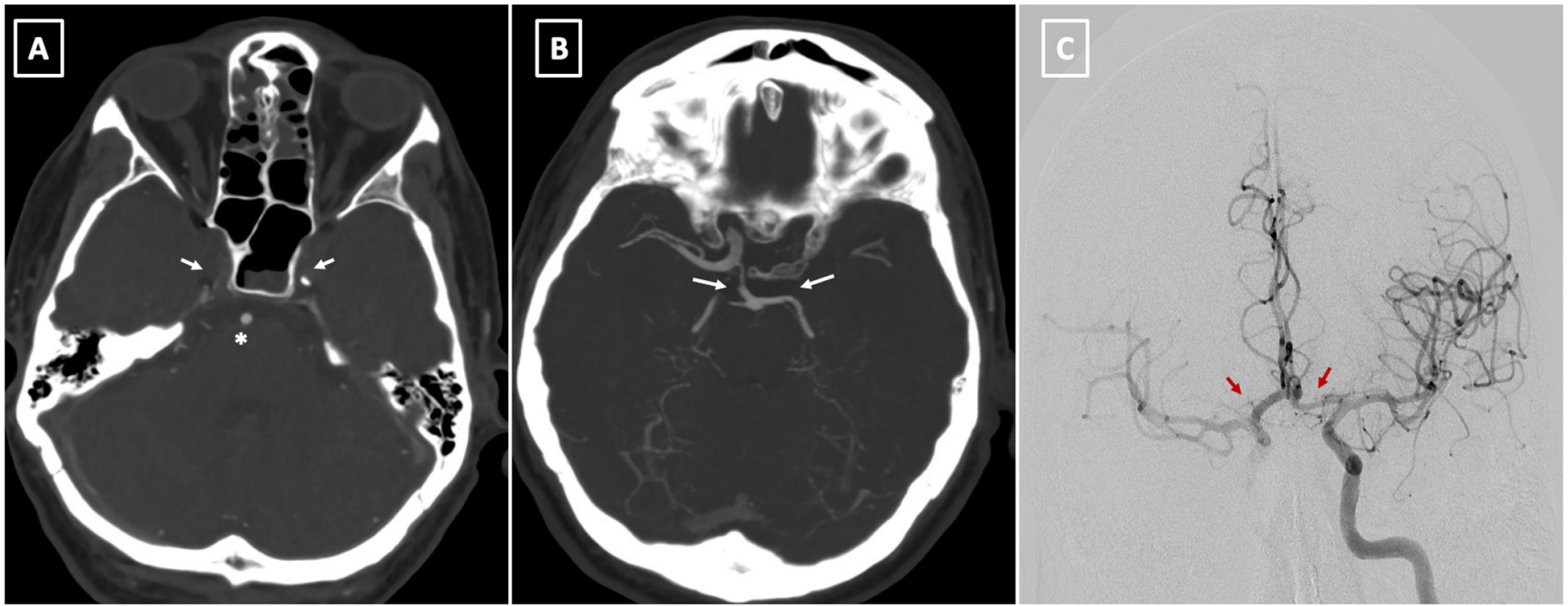
**(A)** preoperative CT angiography at level of skull base demonstrates bilateral occlusion of internal carotid arteries (arrow) with a patent basilar artery (asterisk). **(B)** The Maximal Intensity Projection (MIP) reconstruction highlights the distal filling of the right internal carotid artery through the patent right posterior communicating artery (Pcom). Notably, the left Pcom is absent, resulting in insufficient collateral flow to the left cerebral hemisphere. **(C)** This image shows successful reperfusion of both cerebral hemispheres following the stent placement in the left common carotid artery (LCCA) (arrows).

**Figure 5:**
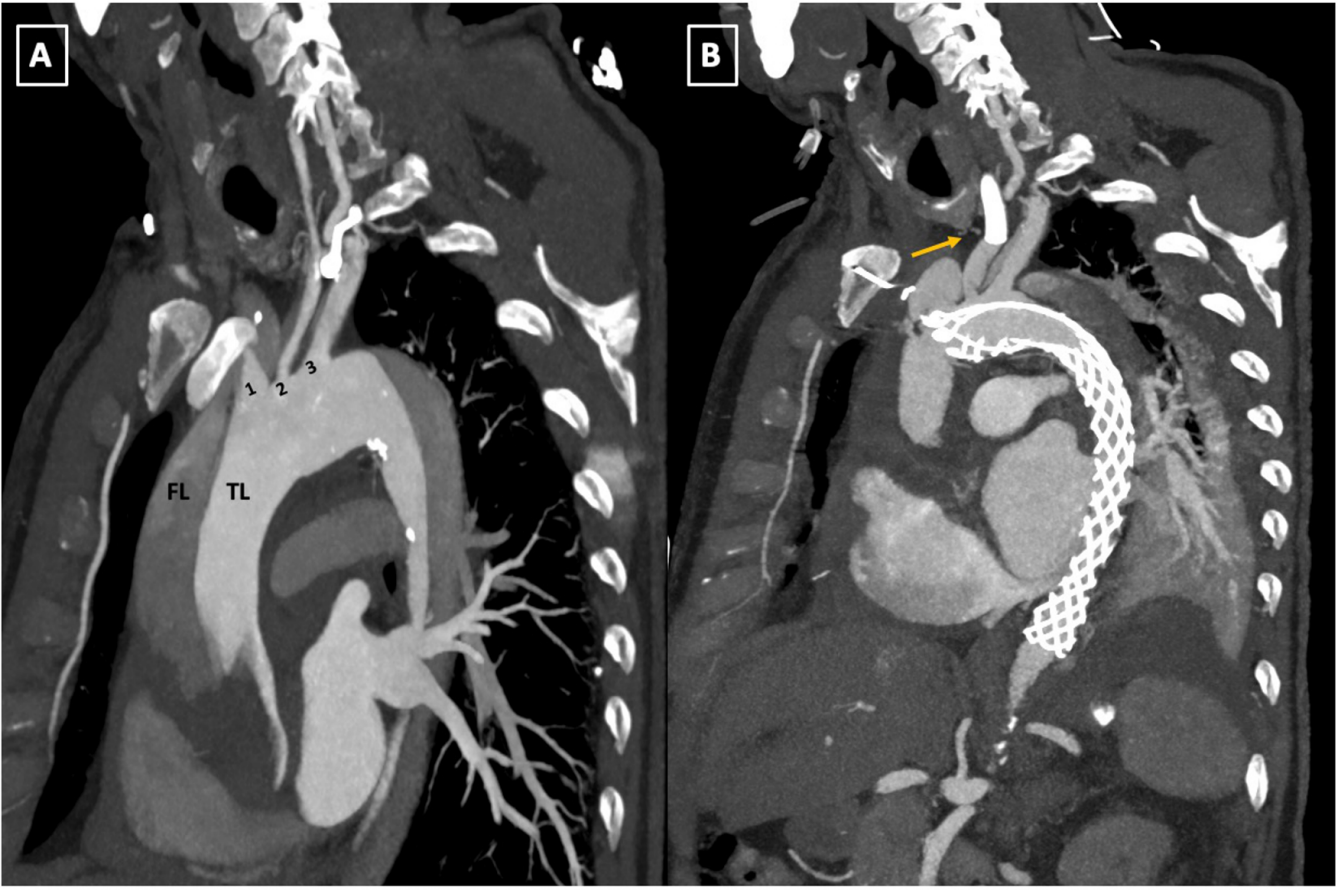
**(A,B**) Pre-and postoperative follow-up computed tomography angiography (CTA) scan comparison. The parasagittal reconstruction view of the aortic arch demonstrates changes placement of the AMDS stent in zone 0 of the aortic arch and left common carotid artery (LCCA) endovascular stent deployment **(**arrow, **B)**. TL, true lumen; FL, false lumen; 1, innominate artery; 2, left common carotid artery; 3, left subclavian artery; AMDS, ascyrus medical dissection stent.

Postoperatively, the patient displayed right anisocoria and aphasia, with cerebral CT identifying bilateral occlusion of internal carotid arteries with a patent basilar artery (Figures 4A,B). Furthermore, the Maximal Intensity Projection (MIP) reconstruction highlights the distal filling of the right internal carotid artery through the patent right posterior communicating artery (Pcom). Notably, the left Pcom is absent, resulting in insufficient collateral flow to the left cerebral hemisphere. The Angiography revealed significant restriction of antegrade flow in the LCCA, markedly diminishing ipsilateral intracranial circulation. Endovascular stenting via the AMDS successfully recanalized the artery. Subsequent angiography post-stent deployment showcased restored patency in the LCCA and markedly improved circulation within the left carotid territory (Figures 4C, 5A,B).

Remarkably, the patient exhibited rapid clinical improvement, nearing neurological baseline swiftly. He was extubated on the first postoperative day (POD) and, after a 15-day ICU stay, was transferred to a regular ward following resolution of post-ischemic psychotic symptoms. The patient was subsequently discharged to a rehabilitation facility on POD 20 in stable condition and without neurological deficits.

## Discussion

The management of ATAAD, particularly when the aortic arch is compromised, remains formidable, with in-hospital mortality escalating to 26% and surging to 40% in the context of end-organ malperfusion (4, 5). The primary objective, often subject to debate, is to ensure patient survival post-surgery while balancing the extent of aortic repair needed against the risk of future arch deterioration.

Within the spectrum of surgical philosophies, ranging from conservative (limited ascending/hemiarch replacement, HAR) to aggressive (total arch replacement, TAR with frozen elephant trunk, FET), no singular procedure is universally optimal. Personalized patient-centric strategies are paramount, emphasizing judicious patient selection as the linchpin of successful outcomes.

Within the spectrum of surgical philosophies, ranging from conservative (limited ascending/hemiarch replacement, HAR) to aggressive (total arch replacement, TAR with frozen elephant trunk, FET), no singular procedure is universally optimal. Personalized patient-centric strategies are paramount, emphasizing judicious patient selection as the linchpin of successful outcomes (7).

Bridging this therapeutic dichotomy, the AMDS heralds an innovative approach in ATAAD treatment. It enhances traditional HAR by incorporating a bare stent into the dissected aortic arch, showing promise in the resolution of SAB-involved dissection post-implantation (8).

Reports by Montagner et al. (9) and Mehdiani et al. (10) demonstrate complete resolution of SAB malperfusion across independent cohorts, with Bozso et al. (11) affirming 95% reperfusion rates of preoperatively compromised vessels, signaling AMDS's potential in obviating the need for extensive TAR reconstruction. Nevertheless, these reparative successes appear uncoupled from neurological sequelae observed post-AMDS intervention.

Nevertheless, these reparative successes appear uncoupled from neurological sequelae observed post-AMDS intervention. Moreover, despite AMDS's less invasive nature, some studies indicate a heightened incidence of postoperative strokes relative to FET in TAR, necessitating critical examination (12).

Unique to AMDS, as an uncovered stent, is its ability to maintain aortic patency by stabilizing the TL without compromising branch vessel integrity or increasing spinal cord injury risk.

In our case, the AMDS facilitated prompt, efficacious endovascular treatment of postoperative cerebral malperfusion via stenting of the SAB, a stroke management strategy not previously documented in ATAAD patients. This capability for immediate endovascular correction underscores AMDS's utility in significantly mitigating cerebral malperfusion consequences following ATAAD.

Recently, the Dissected Aorta Repair Through Stent Implantation (DARTS) trial with AMDS (13) emphasized the imperative of addressing cerebral malperfusion, affecting over half of patients, and revealed a consistent 30-day stroke rate of 15.2%, corroborating the previous literature (14).

At three years, the incidence remains at 17.4%, signaling the need for breakthrough stroke management methods for these patients. Prioritizing refined preoperative diagnostics, rigorous intraoperative monitoring, and meticulous surgical/endovascular techniques will likely improve outcomes. Additionally, comprehensive postoperative care coupled with the pursuit of complementary therapies could reduce risks. Dedicated long-term research is vital for advancing treatment strategies.

## Conclusion

This case is the first of its kind where endovascular stenting through AMDS has successfully resolved a postoperative cerebrovascular insult precipitated by significant malperfusion in LCCA post-ATAAD, marking a potential leap forward in the management of this acute and often lethal condition.

## Data Availability

The original contributions presented in the study are included in the article/Supplementary Material, further inquiries can be directed to the corresponding author.
